# A broadband circularly polarized Fabry Perot antenna with spatially separated superstrate area excitation for CubeSat applications

**DOI:** 10.1038/s41598-023-38440-y

**Published:** 2023-07-11

**Authors:** Ratul De, Mahesh P. Abegaonkar, Ananjan Basu

**Affiliations:** Centre for Applied Research in Electronics, Indian Institute of Technology Delhi, New Delhi, 110016 India

**Keywords:** Electrical and electronic engineering, Engineering

## Abstract

This article describes the development of a high-gain, broadband, circularly polarized Fabry–Perot cavity (FPC) antenna for high-data-rate communication in CubeSat/SmallSat applications. In this work, the concept of spatially separated superstrate area excitation is developed for the first time in FPC antennas. This concept is then validated and applied to increase the gain and the axial ratio bandwidth of a conventional narrowband circularly polarized source patch antenna. The antenna’s design leverages independent control of polarization at different frequencies, resulting in a large overall bandwidth. The fabricated prototype antenna provides right hand circular polarization with a peak measured gain of 15.73 dBic over a common bandwidth of 1.03 GHz ranging from 7.99 GHz to 9.02 GHz. The gain variation over the bandwidth is less than 1.3 dBic. The antenna measures 80 mm × 80 mm × 21.14 mm and is simple, lightweight, easily integrable with CubeSat body, and useful for X-Band data downlink. When integrated with the metallic body of a 1U CubeSat, the simulated gain of the antenna increases to 17.23 dBic, with a peak measured gain of 16.83 dBic. A deployment method is proposed for this antenna that results in a total stowed volume of only 2.13λ_o_ × 2.13λ_o_ × 0.084λ_o_ (0.38 ${\lambda }_{o}^{3}$).

## Introduction

Over the past few years, interest in CubeSats and other classes of small satellites has grown exponentially^1^. They were initially created as tools for training and education, but they are now also capable of carrying out significant scientific tasks^2^. To gather in-situ data from any space object, they are currently used as the secondary payloads of larger satellites or in sacrificial missions^3^. Due to the enormous growth in payload capabilities, they can now downlink huge amount of data quickly, including high resolution imagery and other payload data. High gain and broadband antennas are needed to support these types of data bursts^4^. Additionally, because the CubeSat has a small volume, it may not have very high pointing accuracy since high precision gyros cannot be installed there. So, the orientation of CubeSats in orbit might not be all that stable. As a result, alignment between transmitting and receiving antenna can change and the signal reception may get disrupted. Circularly polarized antennas mitigate the signal degradation due to transmitter–receiver misalignment. Moreover, circularly polarized antennas reduce the effects of polarization mismatches brought on by Faraday rotation in the atmosphere^5^. Therefore, for CubeSat data communications, circularly polarized antennas with a moderate to high gain are preferred.

There have been a lot of high-end antennas developed for CubeSats, and their performance has been very promising. A few of them are the mesh reflector, reflectarray, transmitarray, inflatable, membrane, and metasurface antennas^6,7^. However, the majority of them still have a significant size and are made for larger classes of CubeSats. For smaller classes of CubeSats (size below 2U and nanoSats), choice of antenna for different application is a critical issue. While some applications require highly directional antennas, others call for omnidirectional coverage. High gain antennas with a large CP BW are needed for sacrificial missions or missions involving massive amounts of data transfer. Standard horns that are large and voluminous cannot be used in this situation. Microstrip patch array has low efficiency, a small bandwidth, and feeding losses^8^. In recent years, a number of low profile CubeSat antennas have also been developed which are suitable for small size CubeSats. In^9^, a low profile, wideband CP patch antenna with aperture coupling was suggested. Another S-band patch antenna with a gain of 7.3 dBic was reported for earth observation in^10^. In^11^, a quasi-end-fire SIW antenna with a gain of 13.3 dBi at 18.6 GHz is reported. It radiates in TM_0_ mode from the edge of a grounded dielectric slab. For a 1U CubeSat operating in the frequency range of 27.4–37.3 GHz, a single SIW leaky wave antenna and its array were developed^12^. The antenna offers beam scanning for CP waves. One self-deployable CP Yagi-Uda antenna with a gain of 13.5 dBic was suggested for 1U CubeSats^13^. In^14–16^, other comparable CubeSat antennas are discussed.

Metasurface lens and FPC antennas generally provide high gain and hence can provide a solution for high data rate satellite communication. Various kinds of metasurfaces like zero index^17^, graded index^18^, chiral based metasurface^19,20^ etc. are reported in the literature and they can be utilized for designing high performing lens. Lens antennas generally provide very high gain, however they also have larger dimensions as compared to FPC antennas. As a result FPC antennas are more preferable for small satellite platform and people have recently started using Fabry Perot Cavity (FPC) antennas for high data rate CubeSat communications. The use of a sequentially rotated feed network to obtain wideband CP is proposed for a transparent circularly polarized FPC antenna that is integrated with a solar cell in^21^. However, it has a large structure and a convoluted feeding system. In^22^, a dual band shared aperture circularly polarized antenna is developed. This antenna enables simultaneous communication in the C-band and UHF. The CP bandwidth measured at both frequencies are, however, quite small. Another X-band superstrate antenna with a peak gain of 14.6 dBic over a common BW of 600 MHz is reported in^23^. FPC antennas are small and light-weight, but one of their drawbacks is that typically they have a 0.5λ_0_ height. By using an Artificial Magnetic Conductor (AMC) ground plane, this height can be reduced to 0.25 λ_0_^24^, but AMC ground also results in a smaller antenna bandwidth compared to PEC ground because of its frequency dependence behavior.

There have been many high performing broadband CP FPC antennas reported in the literature, but their high performance comes at the cost of structural complexity^25,26^. As a result, they are suitable for larger satellites and terrestrial operations such as high data rate point to point communication but they are difficult to incorporate in CubeSats. Considering the constraints posed by the small platform, designing high performing FPC antenna for CubeSat application is a challenge. For small satellite applications, the basic requirement is that the antenna should show promising performance with minimal structural complexity. Generally, CP in FPC antennas can be achieved through different techniques like (i) LP source antenna and LP to CP polarization converter^27–30^. In this case, superstrate unit cells are made polarization sensitive which generate two orthogonal E-field component of equal magnitude and 90° phase difference in the transmitted wave thereby producing the CP, (ii) CP source antenna and homogeneous FSS or PRS layer^22,31–34^, (iii) sequentially rotated feed network^35,36^, (iv) receiver-transmitter polarization metasurface^37,38^ etc. However each of these techniques has their own drawbacks, like ^31,33^ have multilayer superstrate and complex basic feed structure, ^29,35^ have low gain, ^30,36–38^ have small common BW and so on. Achieving a broadband CP response with basic CP radiating source is often difficult. In this technique, homogeneous FSS structure with symmetrical unit cells like square, circle, hexagon etc. or uniform dielectric layer is used to enhance the gain while maintaining almost the same state of polarization. As a result, generally if the source radiator is narrowband, the overall FPC antenna will also be narrowband. The only way to improve the CP BW of the antenna then is to make the source broadband; and making the source broadband is not so straightforward and requires special techniques like orthogonal feeding, which eventually increases the design complexity and makes the structure large and bulky as it requires additional power divider and phase shifting network^39^.

This article introduces the concept of SSSA excitation in FPC antennas for the first time and utilizes the same to design a high gain and broadband CP antenna for high data-rate CubeSat communication. Inferences drawn from the parallel plate waveguide theory is used to predict the possibility of SSSA excitation in FPC antennas. It is shown that by managing the resonance conditions in the Fabry–Perot cavity, the antenna can trigger SSSA and thereby govern polarization independently at different frequencies. Despite the narrow CP bandwidth of the primary radiating element, this approach yields a large overall bandwidth and high gain with a very simple structure. Furthermore, this article showcases the integration mechanism of the antenna with 1U CubeSat chassis. A deployment mechanism is also demonstrated for this antenna system that is space- saving for small satellite platforms.

### Theoretical background of spatially separated superstrate area (SSSA) excitation

This section develops the concept of SSSA excitation. The discussion is started with the theory of Modal propagation in parallel plate waveguide, and then it is predicted that SSSA excitation is possible FPC antennas by properly managing the resonance conditions, which eventually allows frequency decoupling.

Any plane electromagnetic wave having electric field vector in the direction $\widehat{n}$, and propagating in any arbitraty direction making an angle ${\theta }_{x}$, ${\theta }_{y},$ ${\theta }_{z}$ w.r.t *x, y,* and *z* axis can be represented as^40^,

1 $$\vec{E} = E\hat{n}e^{{ - j\beta \left( {xcos \theta_{x} + ycos \theta_{y} + zcos \theta_{z} } \right)}}$$

where *β* is phase constant in free space,$\widehat{n}$ is the unit vector and E is the magnitude of the field.

**Figure 1:**
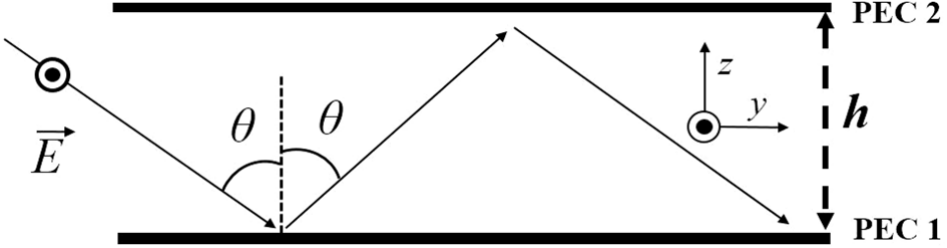
Multiple reflections and modal propagation of EM waves in a parallel plate waveguide.

Figure 1 illustrates that when such a plane wave strikes a 2D parallel plate waveguide at a particular angle *θ*, multiple reflections occur between the two Perfect Electric Conductors (PECs). From Eq. 1, and for the orientation shown in Fig. 1, the incident electric field in PEC 1 can be represented as

2 $$\vec{E}_{i} = E_{1} \hat{x}e^{{j\beta \left( {zcos\theta - ysin\theta } \right)}} \left( {E_{1} {\text{is}}\,{\text{incident}}\,{\text{field}}\,{\text{magnitude}}} \right)$$

And the reflected E field from PEC 1 can be represented as

3 $$\vec{E}_{r} = - E_{1} \hat{x}e^{{j\beta \left( { - zcos\theta - ysin\theta } \right)}}$$

Here, magnitude is negative because of the electric field boundary condition at the PEC.

Total field between the two parallel plates will be then, $\overrightarrow{E}$= ${\overrightarrow{E}}_{i}$+${\overrightarrow{E}}_{r}$.

The superposition of incident and reflected waves results in the formation of standing waves between the two plates and it can be expressed as a generalized expression as

4 $$\vec{E} = A\hat{n}sin\left( {\beta zcos\theta } \right)e^{ - j\beta ysin\theta }$$

where *A* is a constant. In this equation, *Asin(βzcosθ)* represents a standing wave between the two parallel plates and ${e}^{-j\beta ysin\theta }$ represents a travelling wave propagating in the *y* direction. Now we will consider two conditions:

Condition I:

When *θ* = *0°* (normal incidence case), then

5 $$\vec{E} = A\hat{n}sin\left( {\beta z} \right)$$

Which represents a standing wave in the *z* direction. The phase constant in *y* direction is 0, i.e. no wave propagation in the *y* direction ideally.

Condition II:

When *θ ≠ 0*^*o*^ (inclined incidence) and suppose *z* = *h*, then

6 $$\vec{E} = A\hat{n}sin\left( {\beta hcos\theta } \right)e^{ - j\beta ysin\theta }$$

Which means standing waves will be formed between the two plates along the *z* direction, which will propagate in the *y* direction with a phase constant ${\beta }_{y}$ = *βsinθ.* This modal propagation will be sustained when the tangential component of the electric field becomes zero at the PECs, because the boundary condition demands so.

Hence, for sustained modal propagation, *βhcosθ* = *mπ, m* = 1, 2, 3…, *and β* = $\frac{2\pi }{\lambda }$

7 $${\text{So}},{\text{the}}\,{\text{angle}}\,{\text{of}}\,{\text{incidence}}\,\theta = cos^{ - 1} \left( {\frac{m\lambda }{{2h}}} \right),$$

**Figure 2:**
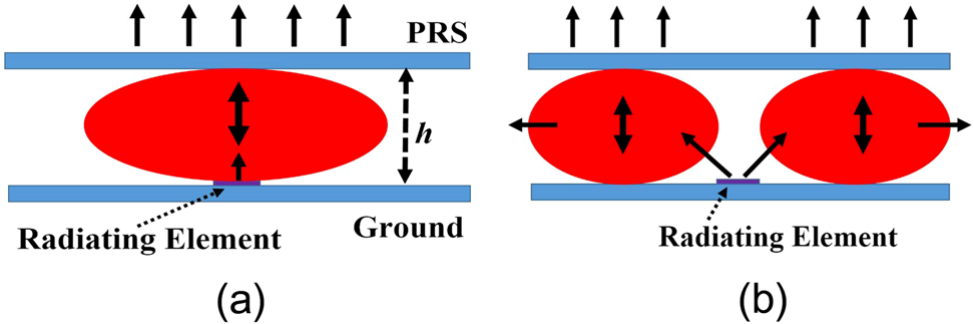
(**a**) Modal pattern at frequency for which resonance condition is satisfied at *θ* = 0°. (**b**) Modal pattern at frequency for which resonance condition is satisfied at *θ* ≠ 0°.

This implies that the waves launched at specific discrete angles will exclusively propagate through the parallel plate waveguide. However, if one of the PECs illustrated in Fig. 1 is transformed into a partially reflecting (and partially transmitting) surface, known as a PRS, then the waves begin to leak through that particular surface. When all the leaked or transmitted waves are in phase, which is the case when resonance condition is satisfied in the cavity, gain enhancement is achieved. If the exciting source or basic radiating element is placed in the cavity formed by the two plates as shown in Fig. 2, then in case of condition I, when the resonance condition is satisfied for normal incidence (when *h* = *nλ/2, n* = 1, 2, 3…), it will give broadside radiation pattern as shown in Fig. 2a. The region of the PRS located just above the radiating element is illuminated because of normal incidence and contributes the most to the broadside radiation. In case of Condition II, the resonance condition is satisfied for inclined angle of incidence, this produces a 2D leaky wave antenna, and the pattern becomes conical for large-sized parallel plates^41^. When the PRS plate size is small, the antenna will not function in 2D leaky wave mode but will act as a 2-element array within a cylindrical region surrounding the source in 3D space, as depicted in Fig. 2b, resulting in a broadside radiation pattern. It is important to note that the PRS region lying immediately above the radiator will not contribute significantly in this case due to the inclined angle of incidence (Fig. 2b). If the transmission coefficient of the PRS is large enough to produce adequate radiation in the broadside direction, the edge radiation, which is responsible for side lobes, will also be minimal. This fundamental concept can be employed in FPC antennas to decouple frequencies. When condition I is satisfied at one frequency and condition II at another, these two frequencies will activate superstrate zones that are spatially distinct (Spatially Separated Superstrate Area, SSSA), allowing for a significant level of design flexibility such as independent control of polarization at these frequencies.

### Validation and application of SSSA excitation

**Figure 3:**
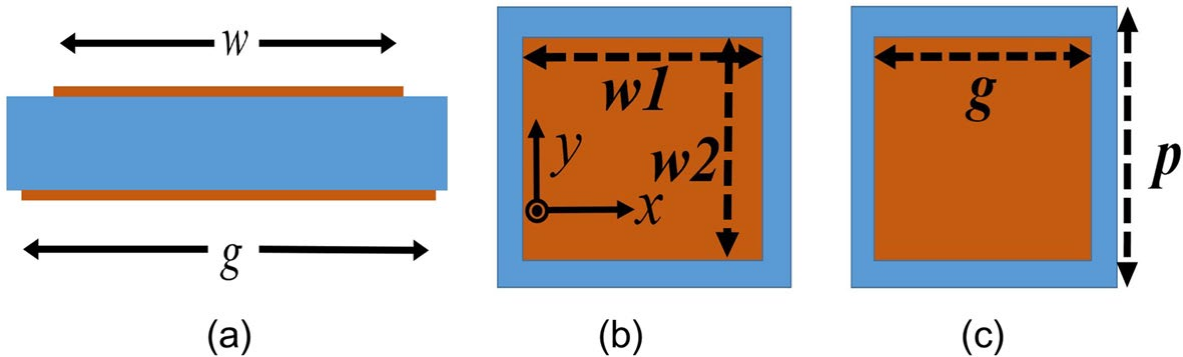
(**a**) Side view of PRS superstrate unit cell. (**b**) Top view. (**c**) Bottom view of superstrate unit cell. Where, *w*1 = *w*2 = *w* = 9 mm, *g* = 10.6 mm, and *p* = 11 mm.

**Figure 4:**
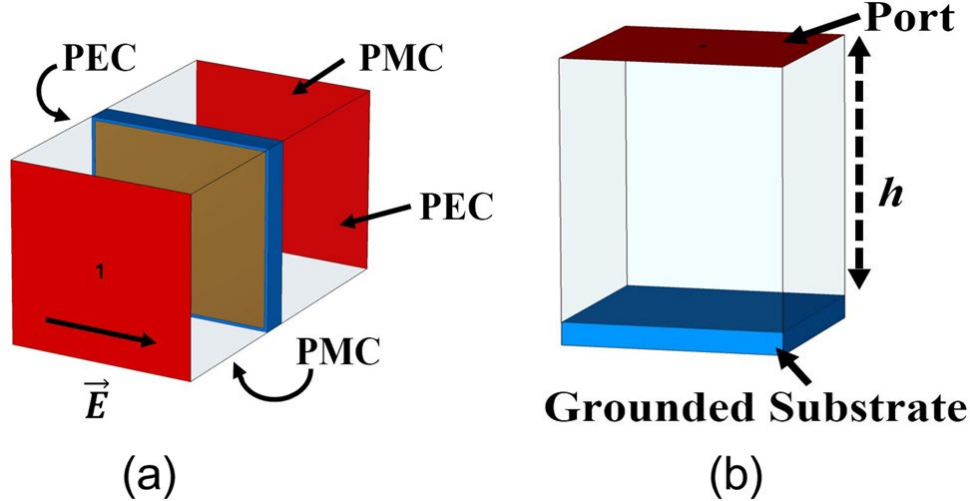
(**a**) Superstrate unit cell under simulation condition. (**b**) Simulation condition for reflection phase due to ground plane + the path length *h*, (*Φ—*2*βh*).

**Figure 5:**
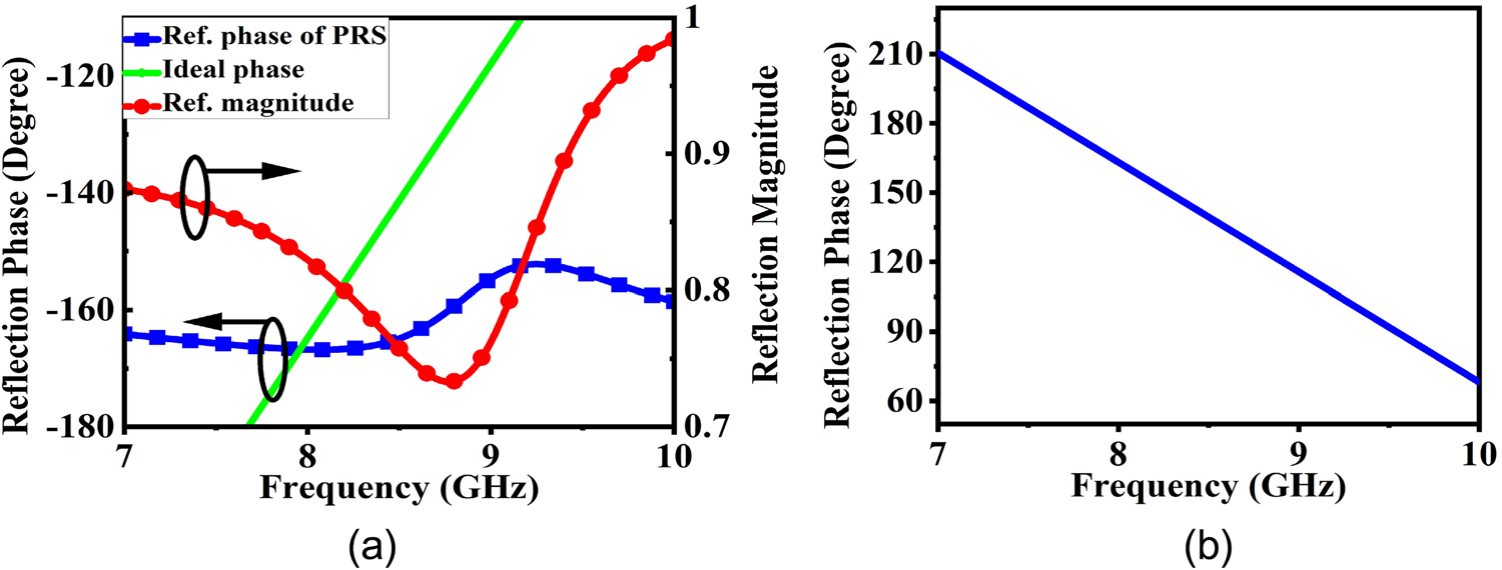
(**a**) Reflection magnitude and phase of PRS superstrate unit cell. (**b**) Reflection phase due to ground plane + the path length *h*, (*Φ—*2*βh*).

This section is dedicated to validating the concept of SSSA excitation at two distinct frequencies. Also, this property can be utilized to design a high gain and broadband CP FPC antenna from a narrowband source. Typically, single feed CP patch antennas have a narrow axial ratio (AR) bandwidth. When operating at frequencies outside this narrow range, the antenna becomes elliptically polarized. However, by adjusting the polarization at these frequencies and bringing the AR slightly below, a wider CP bandwidth can be achieved. This is what we will explore in this section. The condition for SSSA excitation is that, at one frequency, resonance condition should be satisfied for normal incidence and for other frequency, at inclined angle of incidence; and the resonance condition in FPC antenna is related to the superstrate reflection phase. So, the superstrate is designed to provide the desired reflection phase condition for SSSA excitation at both the frequencies. Figure 3 displays different dimensions of the superstrate unit cell, which is composed of a single square dielectric featuring square metallic patches of different size printed on both sides. In Fig. 5a, the superstrate reflection coefficient is presented. By varying the patch dimensions and periodicity of the unit cell, reflection phase of the PRS layer can be adjusted. For a PRS superstrate or FPC antenna, the resonance condition for normal incidence is given by^42^

8 $$\Phi + \varphi - 2\beta h = \pm 2n\pi$$

where ‘*φ*’ is the reflection phase of the PRS and ‘*Φ*’ is the reflection phase of the ground plane, *n* = 0, 1, 2 and ‘*h*’ is the height of the cavity. The increase in directivity due to FPC is given by^42^

9 $$D=10\mathrm{log}\frac{1+\mid R\mid }{1-\mid R\mid }$$

where ‘∣*R*∣’ is the reflection coefficient magnitude. A significant increase in directivity and gain is observed with larger reflections. However, this results in a reduced bandwidth. As a result, a moderate reflection magnitude is typically preferred. Figure 4 illustrates the simulation arrangements utilized for estimating the reflection coefficients of the PRS unit cell, and the reflection phase due to ground plane + the separation distance between the ground and PRS layer *h,* (which is nothing but *Φ—*2*βh*). The simulation employs PEC boundary conditions on two opposite faces of the bounding box and PMC on the other two orthogonal faces, while wave ports are used to stimulate plane waves into the structure. In Fig. 4a, the ports are de-embedded to the PRS surface to obtain the precise reflection phase. From Fig. 5a, it is observed that the magnitude of PRS reflection co-efficient at 8 GHz is 0.822 and at 9 GHz it is 0.76. *‘φ’* is − 166.75° at 8 GHz and − 154.55° at 9 GHz respectively. *Φ—*2*βh* is 163.15° at 8 GHz and 115.8° at 9 GHz as can be seen from Fig. 5b. Based on the information provided in Eq. 8 and the aforementioned data, it is computed that the overall phase delay encountered by the waves upon completing a full reflection cycle (*Φ* + *φ—*2*βh*) within the cavity at 8 GHz is − 3.6°, which is in close proximity to the ideal resonance condition of 0° for normal incidence. As a result, in this instance, condition I outlined in the previous section is met. On the other hand, phase delay at 9 GHz is found to be − 38.75°, so condition II will be satisfied and standing wave will be formed for some inclined angle of incidence and spreads out cylindrically in the cavity. It is important to note that, this concept differs slightly from the conventional positive phase gradient FSS superstrate structures, where the reflection phase is expected to conform to the ideal or optimal phase curve to ensure proper resonance conditions for normal incidence across all operating frequencies^43–45^. In the case of SSSA excitation, however, it is desirable to achieve resonance conditions for normal incidence at one frequency and for inclined incidence at another frequency. Equation 8 can be rewritten as

10 $$\varphi = \frac{4\pi hf}{c} \pm \left( {2n - 1} \right)\pi$$

where *f* is the frequency of operation, *c* is the speed of light in free space, and *Φ* = π for PEC ground. This equation predicts the ideal phase values (plotted in Fig. 5a) for proper resonance at normal incidence in the cavity. It is important to note that in our case, the PRS phase intersects the ideal phase plot at only one frequency, which is 8 GHz. This implies that the resonance condition will only be satisfied for normal incidence at 8 GHz. For other frequencies, the resonance condition will be satisfied for inclined incidence, as per the theory discussed in the previous section.

**Figure 6:**
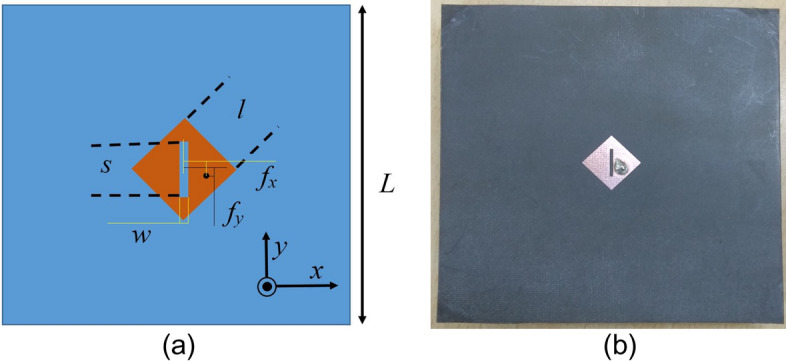
(**a**) Basic CP source patch, *l* = 9.9 mm, *s* = 7 mm, *w* = 0.8 mm, *fx* = 2.6 mm, *fy* = 1.4 mm, *L* = 80 mm. (**b**) Fabricated prototype.

**Figure 7:**
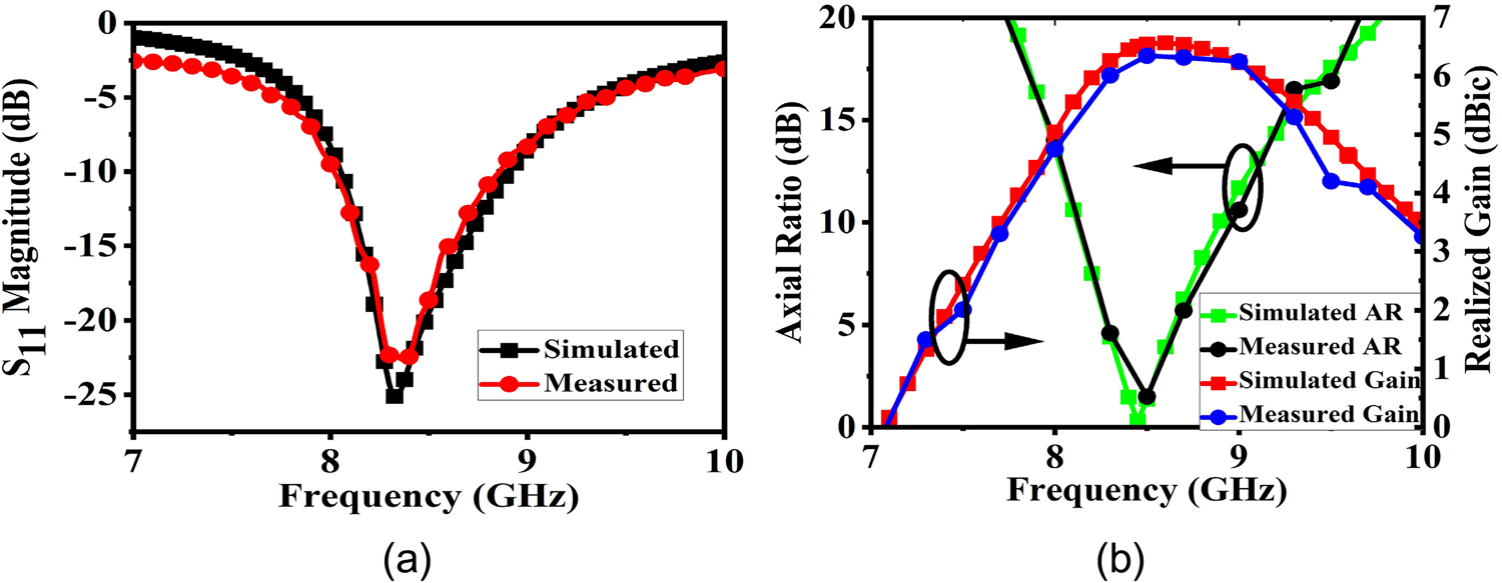
(**a**) S_11_ magnitude of the source CP patch. (**b**) AR and realized gain plot of source patch.

**Figure 8:**
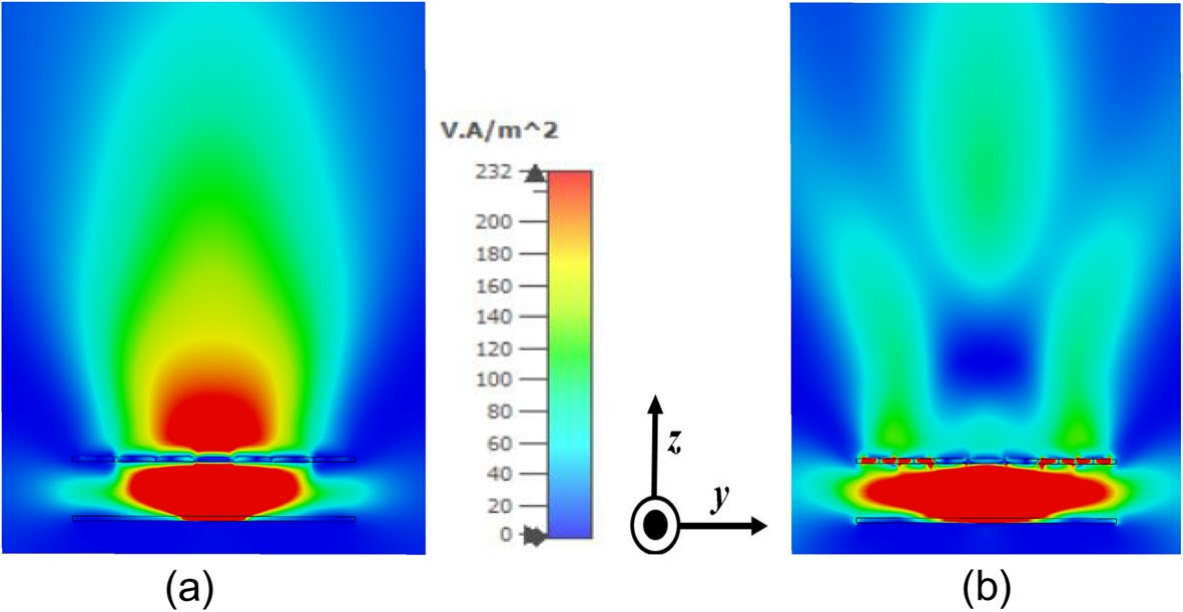
(**a**) Power flow distribution along the cut section of the antenna at 8 GHz. (**b**) Power flow distribution at 9 GHz.

**Figure 9:**
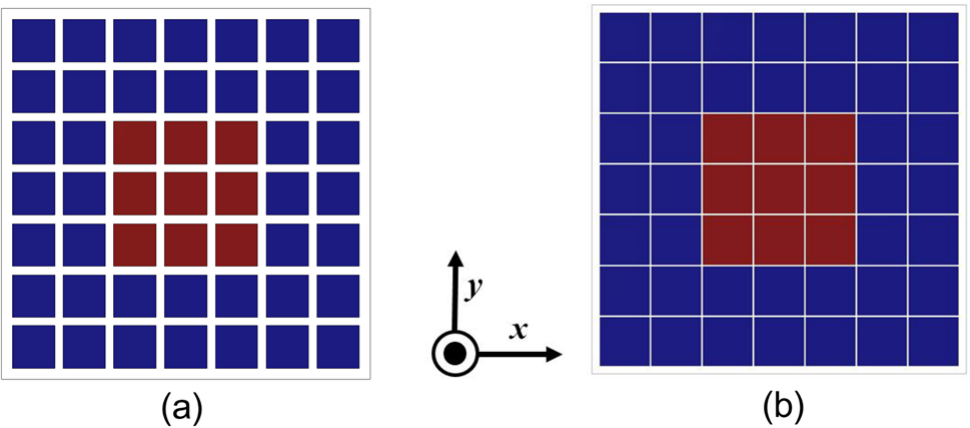
(**a**) Top view of PRS superstrate. (**b**) Bottom view of the superstrate. Central red patches are excited at 8 GHz and the outer blue patches at 9 GHz due to SSSA excitation.

An array of 7 × 7 such symmetrical unit element is used to form the uniform superstrate, and a CP patch antenna designed at around 8.5 GHz as shown in Fig. 6 is used to feed the Fabry–Perot cavity. The responses of the feed antenna are shown in Fig. 7. As shown in Fig. 7b, the axial ratio (AR) at 8.5 GHz is below 3 dB, but it is 13.57 dB and 11.68 dB at 8 GHz and 9 GHz, respectively. This radiating source has an impedance bandwidth of 850 MHz, a 3 dB AR bandwidth of 148 MHz, and a gain of 6.55 dBic. The FPC antenna lies in the x- y plane, and the power flow diagrams at both 8 and 9 GHz are shown in Fig. 8. The power flow diagram indicates that at 8 GHz, the central part of the superstrate is the main contributor to the broadside radiation due to normal resonance condition, while at 9 GHz, the outer peripheral area contributes the most because of inclined resonance. Figure 9 displays the superstrate structure with colour coding representing the patches that are being impacted or getting illuminated at both the frequencies due to SSSA excitation. It can be concluded from Fig. 8b that at 9 GHz, the structure acts like a two-element array in both the orthogonal planes (*xz* and *yz*) due to its symmetry, and the fields add up in the far field to increase the gain in the broadside direction. The above discussion validates the proposed concept of SSSA excitation in FPC antennas.

**Figure 10:**
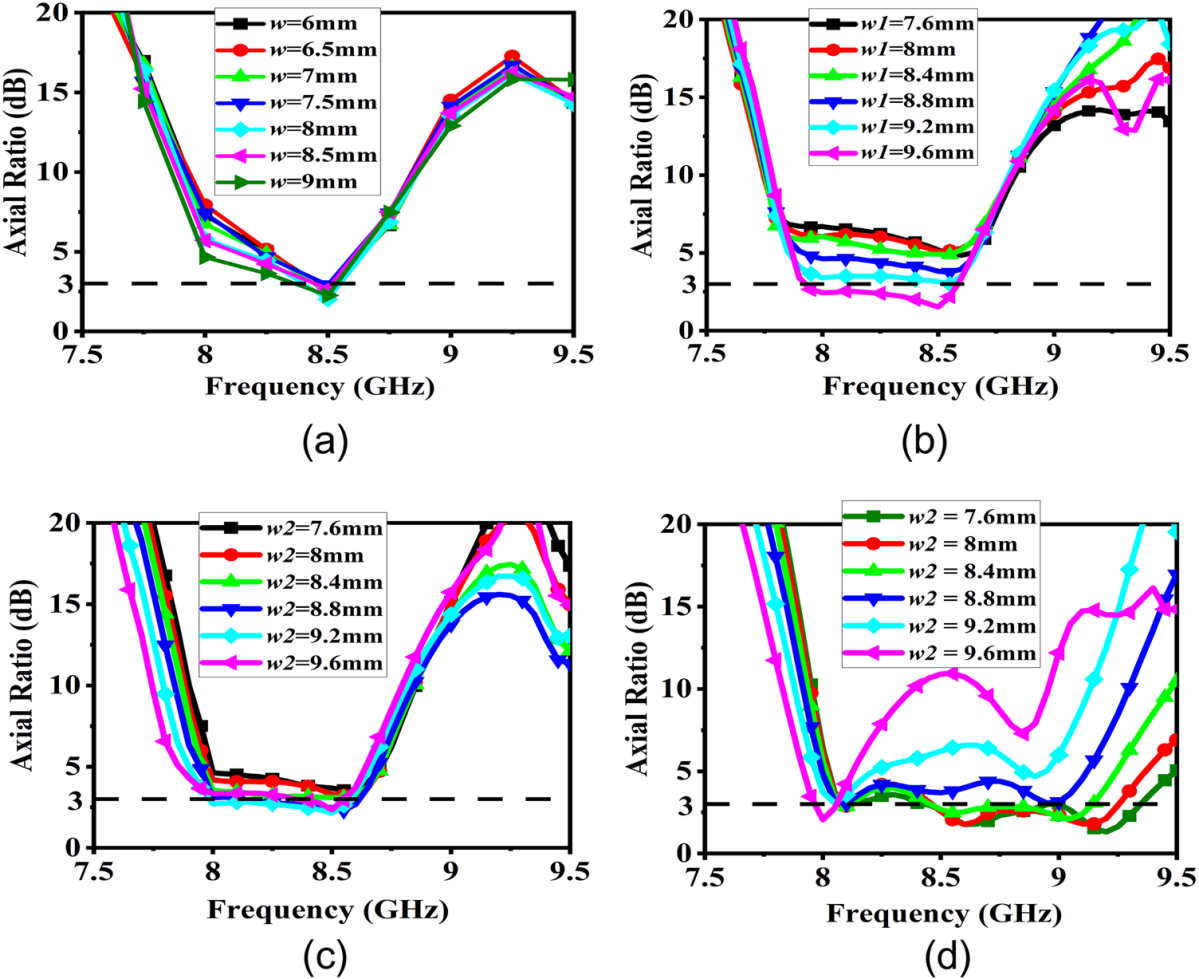
(**a**) Parametric study of the upper patch size variation of the superstrate with *g* = 10.6 mm and *p* = 11 mm. *w*1 = *w*2 = *w*. (**b**) Parametric study of upper central patch size variation of the superstrate with *w*2 = 9.4 mm. (**c**) Parametric study of upper central patch size variation with *w*1 = 9.4 mm. (**d**) Parametric study of upper outer patch size variation of the superstrate with *w*1 = 9.4 mm.

**Figure 11:**
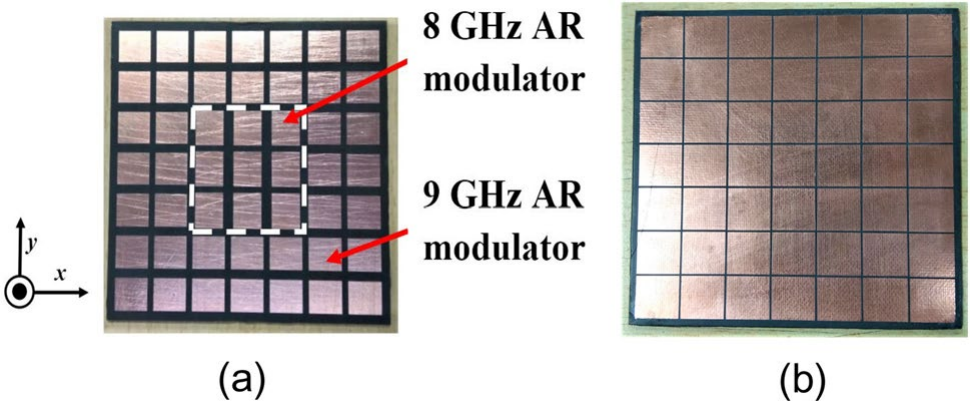
(**a**) Top view of the modulated PRS superstrate. (**b**) Bottom view.

**Figure 12:**
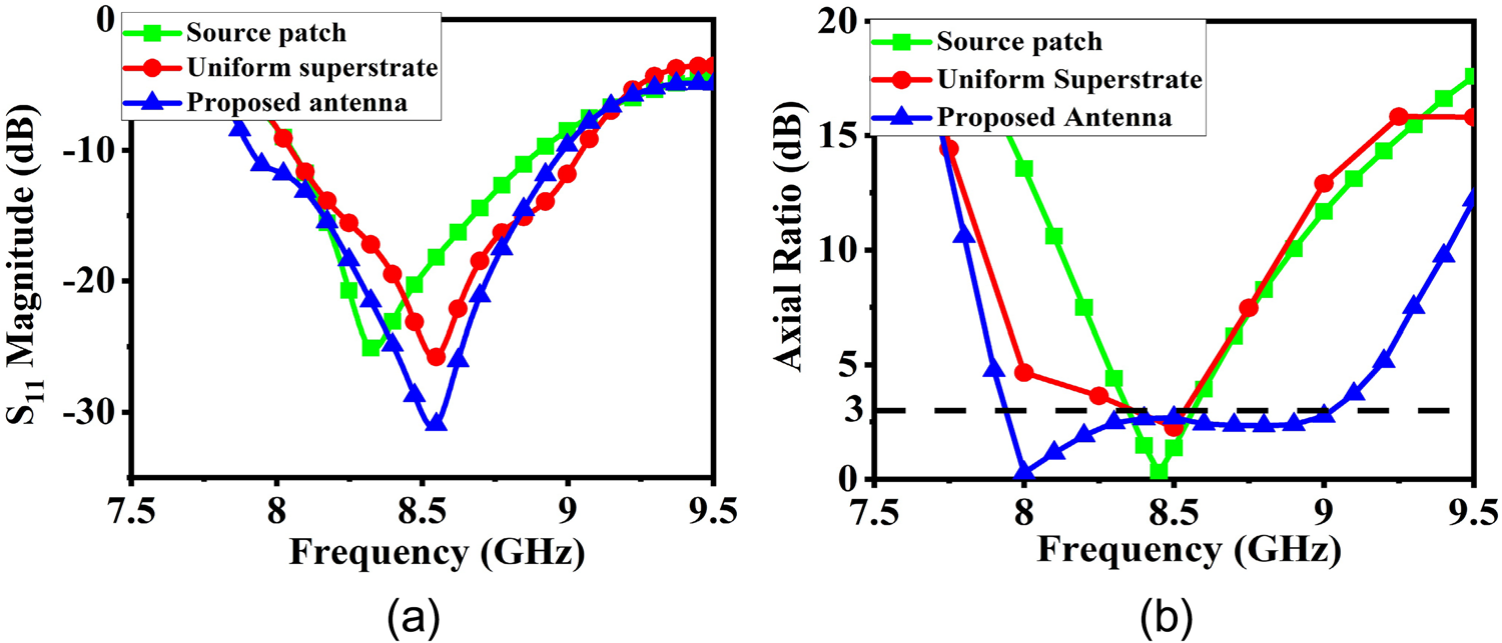
(**a**) S_11_ magnitude comparison of three CP cases. (**b**) AR comparison of three CP cases.

With uniform symmetrical unit cells, however, it is not possible to achieve a broadband CP performance although the gain is enhanced over the band. A parametric study of uniform superstrate unit cell size variation is shown in Fig. 10a and it clearly shows the CP BW is quite small in this case. To address this issue, the patch dimensions of the superstrate are modified to make them polarization-sensitive and to provide different responses to the two orthogonal components of the electric fields. Furthermore, according to the resonance conditions, the central patches of the superstrate would be excited at 8 GHz, and the outer patches would be excited at 9 GHz, respectively. By manipulating the dimensions of the upper patches of the superstrate, it is possible to alter the polarization state of the antenna, and the SSSA excitation provides the flexibility to control the AR independently at different frequencies. When the dimensions of the upper inner patches of the superstrate are changed, the AR of the antenna begins to shift at 8 GHz, and at certain dimensions, pure CP is obtained. The same is observed for the outer patches at 9 GHz. A parametric study of the superstrate upper patch dimension change is presented in Fig. 10b–d. In this study, when considering 8 GHz AR modulation, only upper central patches of the superstrate were present and the outer patches responsible for 9 GHz AR modulation have been removed and vice-versa while keeping the bottom patches of the superstrate intact with *p* = 11 mm and *g* = 10.6 mm. It is found that when both modulating patches are present with optimized dimensions, the structure provides a wide AR BW of around 1 GHz while achieving high gain enhancement across the whole band. The final modulated superstrate is shown in Fig. 11 and the optimized superstrate patch dimensions are: for central patches *w*1 = 8.2 mm, *w*2 = 9.4 mm, and for outer patches *w*1 = 9.4 mm, *w*2 = 8.6 mm, *p* = 11 mm, and *g* = 10.6 mm. A comparison between the S_11_ magnitude and AR plots of the three cases (the basic radiating patch, superstrate with uniform unit cell and the FPC antenna with modulated PRS) are shown in Fig. 12 and clearly shows the viability of SSSA excitation in FPC antennas. It should be noted that the elliptical polarization may occur due to the unequal magnitude of the two orthogonal components of the electric field with a ± 90° phase difference or due to some other phase difference between two equal orthogonal components of electric field. This method can be used to bring the AR below 3 dB if it is not too high.

### Experimental results and discussions

**Figure 13:**
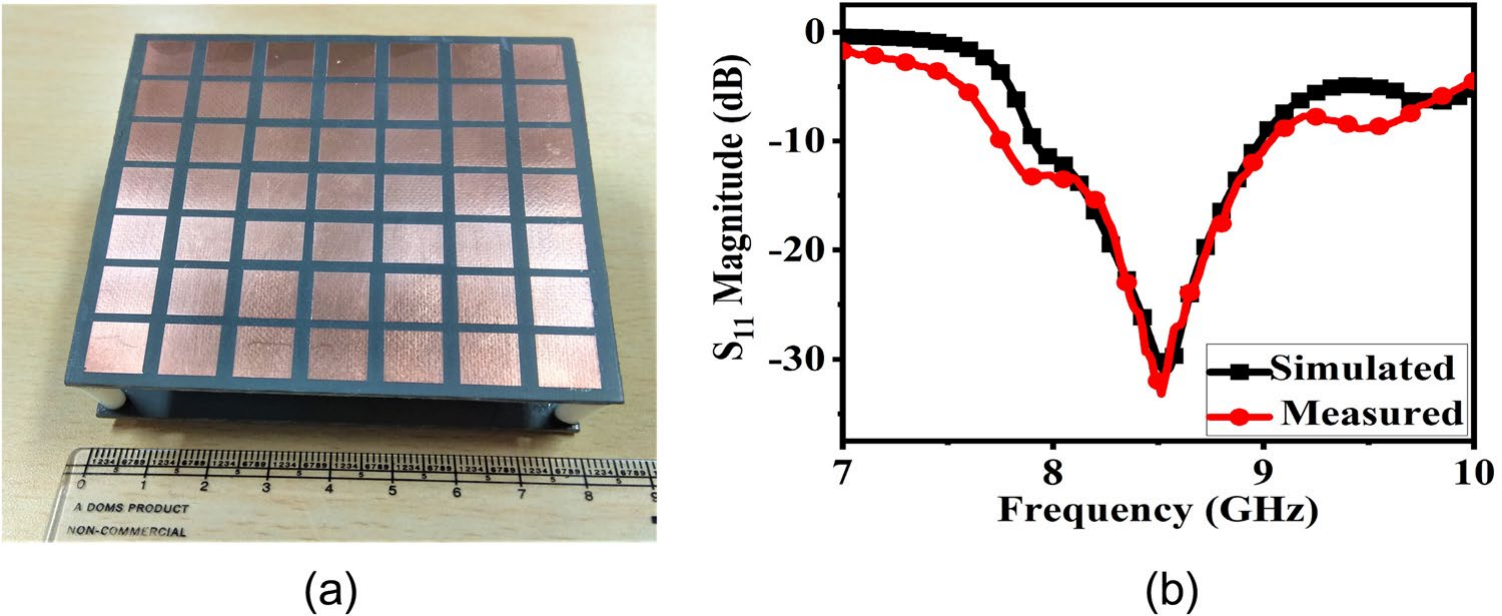
(**a**) Fabricated prototype antenna. (**b**) Comparison of S_11_ magnitude response of the fabricated antenna with the simulation.

**Figure 14:**
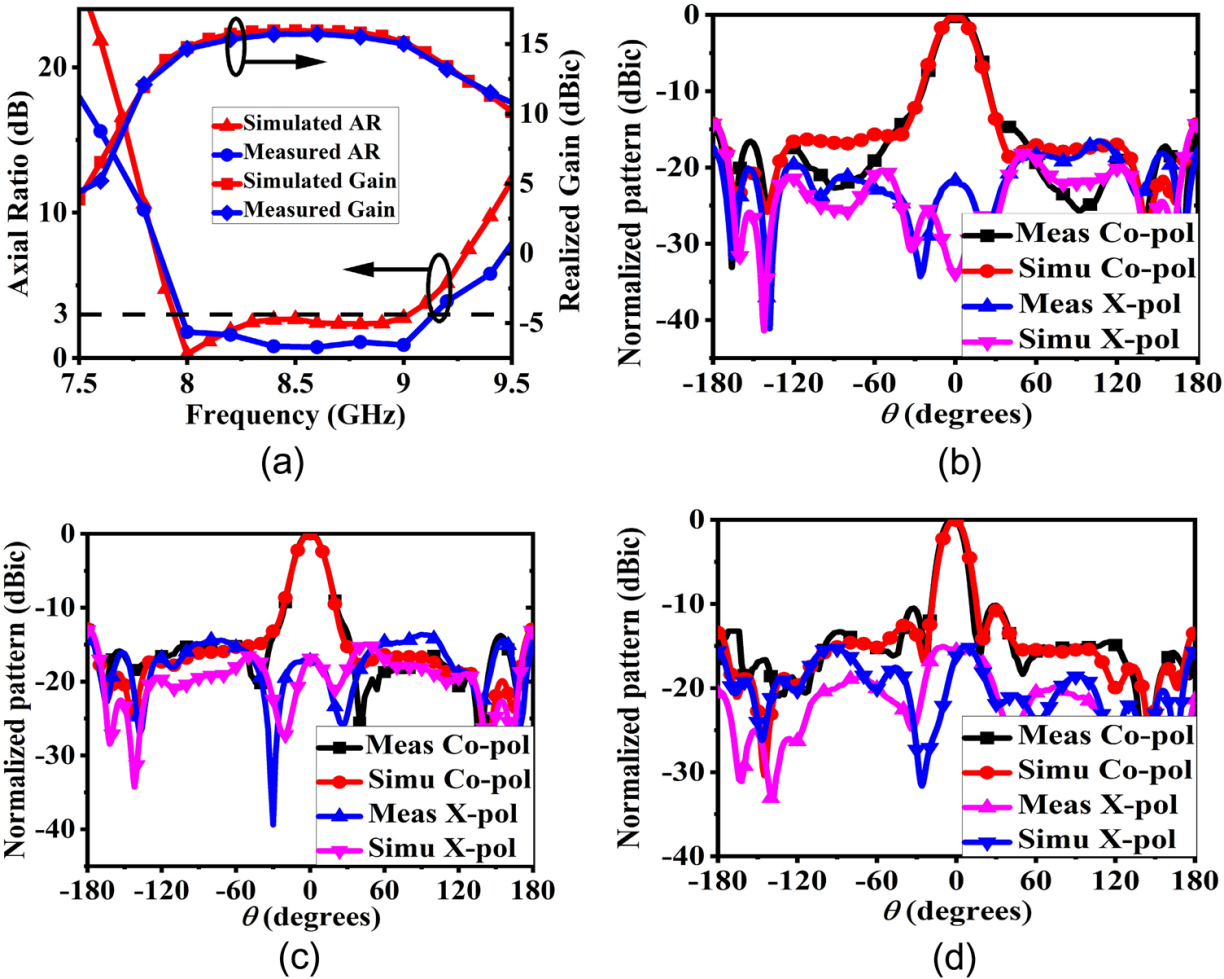
(**a**) Gain and AR comparison of the prototype antenna with the simulation. (**b**) Normalized pattern at 8 GHz. (**c**) Normalized pattern at 8.5 GHz. (**d**) Normalized pattern at 9 GHz.

**Figure 15:**
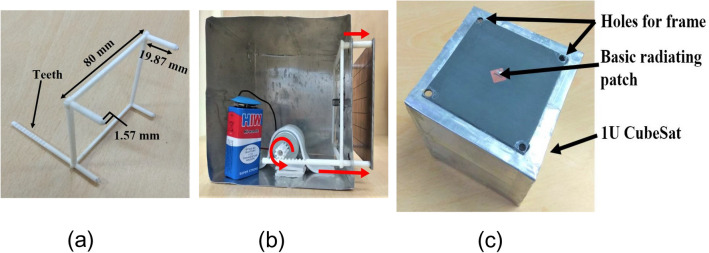
(**a**) 3D printed frame. (**b**) Deployment mechanism of the antenna with frame and DC micro-motor. (**c**) Basic source CP patch and ground plane mounted on CubeSat body.

**Figure 16:**
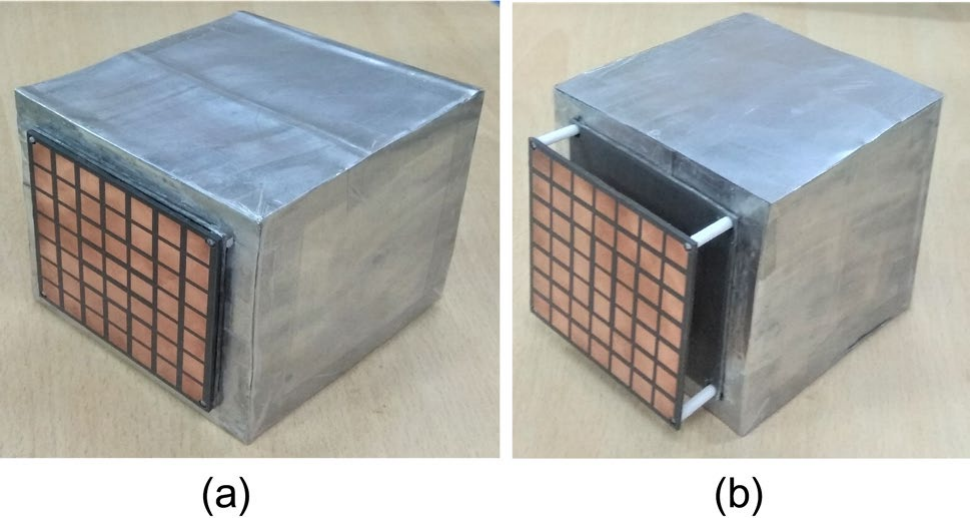
(**a**) Stowed antenna on CubeSat body. (**b**) Fully deployed antenna.

**Figure 17:**
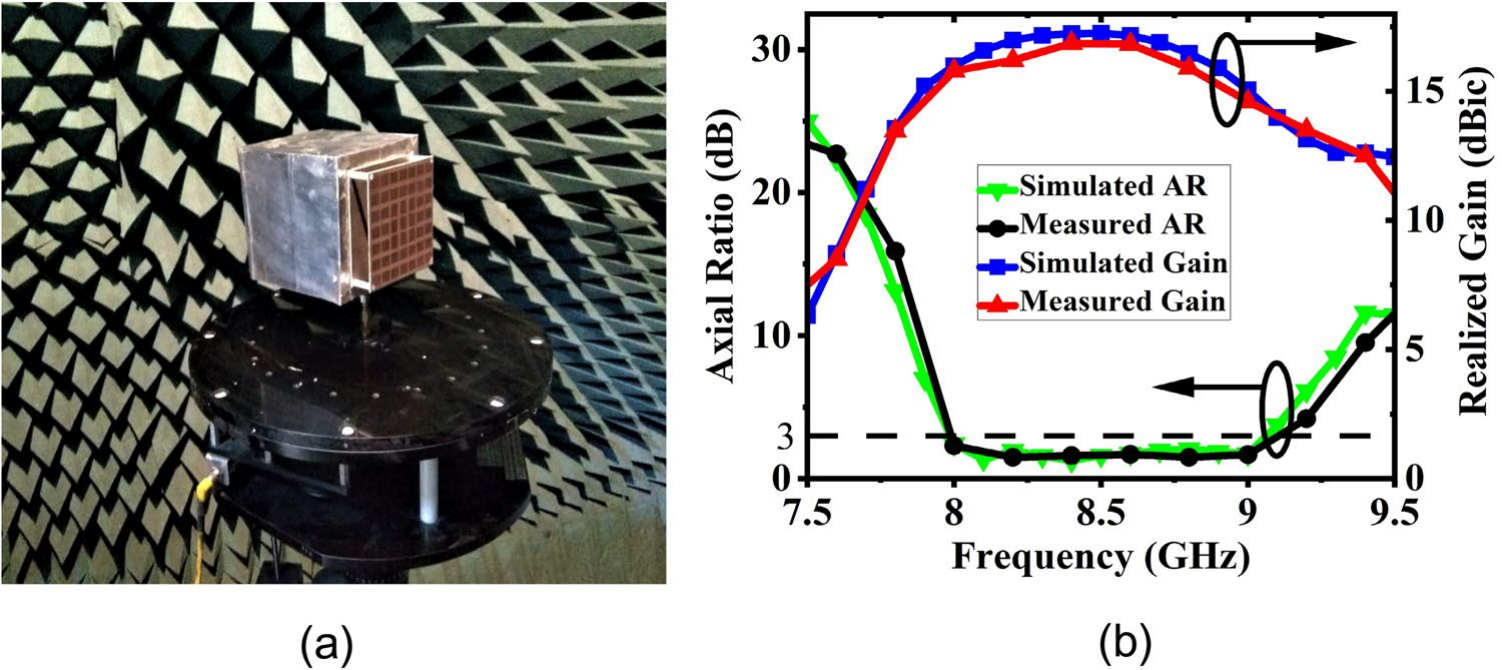
(**a**) Final prototype antenna installed on a 1U CubeSat in a measurement setup. (**b**) Gain and AR comparison of the final fabricated antenna mounted on the 1U CubeSat.

The final broadband CP FPC antenna is simulated in CST microwave studio software and a prototype is fabricated using standard photolithography process on Rogers RT Duroid 5880 substrate with a dielectric constant of 2.2 and a loss tangent of 0.001. Rogers RT/Duroid 5880 is a space qualified substrate which has passed various space material tests^46^ like material outgassing, temperature fluctuations, and radiation exposure. As a result, they are used extensively for space applications^4^. To construct the superstrate, a 7 × 7 array of modulated unit cell is utilized with both the superstrate and the ground plane measuring 80 mm × 80 mm and having a thickness of 1.57 mm. The superstrate is elevated at a height of h = 18 mm using four Teflon posts, which is close to λ_o_/2 at 8 GHz. The prototype is shown in Fig. 13a, with its responses displayed in Figs. 13b and 14. The S_11_ is measured using Agilent PNA network analyzer E8364C, and pattern is measured in an anechoic chamber. The simulated impedance BW is 1.08 GHz, ranging from 7.92 to 9 GHz, while the measured BW is 1.26 GHz (7.76–9.02 GHz). Figure 14a shows the measured and simulated realized gain and AR. It can be observed that the AR is staying below 3 dB for a BW of 1.135 GHz (7.99–9.125 GHz). The measured peak gain is 15.73 dBic with almost a flat response. In the entire operating band, the gain variation is less than 1.3 dBic. The antenna provides RHCP, with X-Pol level below − 16 dB in the broadside direction. Normalized patterns are shown at 8 GHz, 8.5 GHz, and 9 GHz in Fig. 14b, c, d respectively. Small side-lobes are observed at 9 GHz due to power leakage from edges of the antenna at this frequency, however side-lobe level is below − 10 dB and will not degrade CubeSat performance. Little discrepancy between the simulated and measured data is attributed to fabrication tolerances and measurement errors. The antenna can be installed on CubeSat for high data-rate downlinks, but it’s height is approximately 0.5λ_o_ and occupies an effective CubeSat volume of 80 mm × 80 mm × 21.14 mm, which is quite large for a smaller CubeSat (size < 2U). A significant amount of space can be saved by employing a deployment mechanism, which works on the principle of rack and pinion. For that, a frame is made by 3D printing as shown in Fig. 15a. Teeth are created on one of the legs of the frame and a DC micro-motor is used to move the frame back and forth (Fig. 15b). The superstrate is attached to the moving frame, whereas the ground plane and the basic radiating patch are mounted on the fixed CubeSat metallic body as shown in Fig. 15c. Four holes are created on the CubeSat metallic body and the corners of the grounded substrate, through which the frame could slide through. Before launch, the antenna could be stowed on the CubeSat face and once it is in the orbit, the antenna could be deployed as shown in Fig. 16 by using a trigger in the micro-motor. It is observed that when the antenna is installed on 1U CubeSat metallic body, then the simulated maximum gain increases to 17.23 dBic due to ground reflection, however AR performance degrades little bit which can be rectified by optimizing the superstrate patch size further. The final optimized dimensions are: for central patches *w*1 = 8.2 mm, *w*2 = 9.4 mm, and for outer patches *w*1 = 9.6 mm, *w*2 = 8.4 mm.* p* = 11 mm, and *g* = 10.6 mm. A superstrate is fabricated with these dimensions on Rogers 5880 substrate, and the final antenna is integrated with 1U CubeSat and measured (Fig. 17a). The integrated antenna shows a measured maximum gain of 16.83 dBic with AR below 3 dB over the band as shown in Fig. 17b with negligible effect on S_11_. A comparison table with the circularly polarized high gain state of the art antennas recently developed for CubeSat (size < 2U) applications is given in Table 1, which shows the promising performance of the proposed antenna. From the table, it is clear that the proposed antenna has the maximum gain and maximum common BW with minimum stowage volume requirement. Little side lobe appears at 9 GHz, but at other frequencies, side lobes are not there. Because of this great performance, the proposed antenna is a strong candidate for small satellite payload data transfer applications.

**Table 1:** Comparison of the proposed work with other similar CP high gain CubeSat (size < 2U) antennas.

Refs.	Common fractional bandwidth (FBW %)	Peak gain (dBic)	Stowed volume	Side lobe level	Cross Pol discrimination (XPD)	Configuration	Deployable
^13^	5.6–6 GHz (6.9%)	13.5	1.93λo × 1.93λo × 0.31λo (1.15 ${\lambda }_{o}^{3}$)	− 15 dB	> 15 dB	Stacked Yagi-Uda	Yes
^16^	5.45–5.55 GHz (1.8%)	12.4	4.22λo × 1.93λo × 0.03λo (0.24 ${\lambda }_{o}^{3}$)	− 12 dB	–	Aperture coupled patch	No
^22^	5.72–5.8 GHz (1.4%)	13.8	1.93λo × 1.93λo × 0.75λo (2.79 ${\lambda }_{o}^{3}$)	− 12 dB	> 15 dB	Fabry Perot resonator	No
^23^	8.28–8.88 GHz (7%)	14.6	1.77λo × 1.77λo × 0.64λo (2.01 ${\lambda }_{o}^{3}$)	− 14.8 dB	> 27.3 dB	Partially reflecting surface	No
This work	7.99–9.02 GHz (12.11%)	15.73 standalone and 16.83 when installed on 1U CubeSat	2.13λo × 2.13λo × 0.084λo (0.38 ${\lambda }_{o}^{3}$)	− 11.2 dB	> 16 dB	FPC with SSSA excitation	Yes

## Conclusion

This article presents a broadband, high gain, circularly polarized FPC antenna for high data-rate CubeSat/SmallSat communications. The design is based on the SSSA excitation concept, which is derived from the parallel plate waveguide theory, and applied to the FPC antennas for the first time. The SSSA excitation technique allows for different superstrate areas to be excited at different frequencies, enabling the control of AR independently. This results in a broadband and high gain CP FPC antenna from a narrowband source radiator. The standalone antenna has a 1.03 GHz common bandwidth starting from 7.99 GHz to 9.02 GHz with a measured peak realized gain of 15.73 dBic. When mounted on a CubeSat body, the measured gain increases to 16.83 dBic due to ground reflection. A deployment mechanism is also demonstrated, which allows the antenna to be stowed in a compact size, saving valuable space.

## Data Availability

All the available data are inherent in this manuscript. There are no supplementary material.
